# International Adoption of Children with Special Needs in Spain

**DOI:** 10.3390/children10040690

**Published:** 2023-04-05

**Authors:** Alicia Hernanz Lobo, Arantxa Berzosa Sánchez, Lucía Escolano, Sara Pérez Muñoz, Nathalia Gerig, Talía Sainz, María Jose Mellado Peña, Milagros García López Hortelano

**Affiliations:** 1 Pediatric Infectious Diseases Department, Gregorio Marañón University Hospital, 28007 Madrid, Spain; 2 Gregorio Marañón Research Health Institute (IiSGM), Translational Research in Pediatric Infectious Diseases (RITIP), 28007 Madrid, Spain; 3 Centro de Investigación Biomédica en Red de Enfermedades Infecciosas (CIBERINFECC), Instituto de Salud Carlos III, 28029 Madrid, Spain; 4 Medicine Faculty, Complutense University of Madrid, 28040 Madrid, Spain; 5 Department of Pediatrics, Clínico San Carlos Hospital, Clínico San Carlos Research Health Institute (IdISSC), 28040 Madrid, Spain; 6 Department of Pediatrics, La Paz University Hospital, 28046 Madrid, Spain; 7 Department of Pediatrics, Torrejon Hospital, 28850 Madrid, Spain; 8 Department of Pediatrics, Infectious and Tropical Diseases, Paediatrics, La Paz University Hospital, Paseo de la Castellana, 261, 28046 Madrid, Spain; 9 La Paz Hospital and La Paz Research Institute (IdiPAZ), Translational Research in Pediatric Infectious Diseases (RITIP), 28046 Madrid, Spain; 10 Medicine Faculty, Autónoma University of Madrid, 28049 Madrid, Spain

**Keywords:** adoptees, microcephaly, delay in pondero-statural growth

## Abstract

International adoption has declined in recent years, although the adoption of children with special needs has arisen. We aim to describe our experience in the international adoption of children with special needs and to analyze the concordance between the pathologies included in pre-adoption reports and the diagnosis made upon arrival. We conducted a retrospective descriptive study including internationally adopted children with special needs evaluated at a reference Spanish unit between 2016 and 2019. Epidemiological and clinical variables were collected from medical records, and pre-adoption reports were compared to established diagnoses following their evaluation and complementary tests. Fifty-seven children were included: 36.8% females, a median age of 27 months [IQR:17–39], mostly coming from China (63.2%) and Vietnam (31.6%). The main pathologies described in the pre-adoption reports were congenital surgical malformations (40.3%), hematological (22.6%), and neurological (24.6%). The initial diagnosis that motivated the international adoption via special needs was confirmed in 79% of the children. After evaluation, 14% were diagnosed with weight and growth delay, and 17.5% with microcephaly, not previously reported. Infectious diseases were also prevalent (29.8%). According to our series, the pre-adoption reports of children with special needs appear accurate, with a low rate of new diagnoses. Pre-existing conditions were confirmed in almost 80% of cases.

## 1. Introduction

International adoptions (IAs) have declined sharply in recent years. Socioeconomic conditions in many regions have improved. Furthermore, according to international recommendations by the World Health Organization and other institutions, foster families within the country of origin are preferred over IA in order to minimize the adaptation process. The requirements to complete this complex procedure have been tightened: many countries have limited the profile of adopters and, concurrently, reception countries, including Spain, have limited adoptions from countries that still need to guarantee an entirely safe process. In 2015, 801 IAs were performed in Spain, compared to 370 in 2019. Of the latter, 65.4% were from Asia, 19.5% from European countries, 14% from Latin America, and 1.1% from Africa [1].

With the general decline in number, the proportion of children adopted presenting pre-existing conditions has increased in Spain and other countries, such as France and the United States [2,3]. The definition of “special needs” (SN) arises from the urgency of speeding up the adoption of minors with chronic pathologies requiring evaluation and treatment in a short period [4]. Heart defects, orthopedic problems, infectious diseases, or neurodevelopmental disorders are among the most prevalent diseases. In China, children presenting with SN can be internationally adopted via a fast track called “green passage”. In Vietnam, those children are adopted via “list 2”.

International adoptions represent an excellent opportunity for children presenting with medical conditions born in resource-limited settings. However, the success of the process requires a well-informed adoptive family and an experienced and supportive professional team. During the pre-adoption process, referral units offer the possibility of discussing each situation's clinical implications to facilitate the foster parents' understanding of the child’s condition, healthcare requirements, follow-up, treatment needs, and quality of life in the short and long term. After arrival, a complete medical exam, including anthropometric measurements, imported diseases screening, and physical and psychological evaluation, is performed to confirm the previous diagnosis, optimize treatment and adjust the immunization schedule. With the appropriate care, most children achieve a high quality of life. However, the complex bureaucratic process and adaptation imply significant challenges and emotional costs, and pre-existing conditions undoubtedly generate uncertainty. Surprisingly, data regarding the unique population of internationally adopted children with special needs are scarce.

This study aims to describe the epidemiological and clinical characteristics of a cohort of internationally adopted children with SN. We aim to analyze the discrepancies, if any, between the pre-adoption reports provided during the adoption process and the clinical findings after a thorough evaluation in a referral unit over a three-year period.

## 2. Materials and Methods

### 2.1. Study Design

A retrospective descriptive study was designed and performed at a referral unit at Children’s Hospital La Paz., Madrid, including all internationally adopted children with SN (particularly called “green passage” if they came from China or “list 2” from Vietnam), who were evaluated between January 2016 and July 2019.

**Figure 1:**
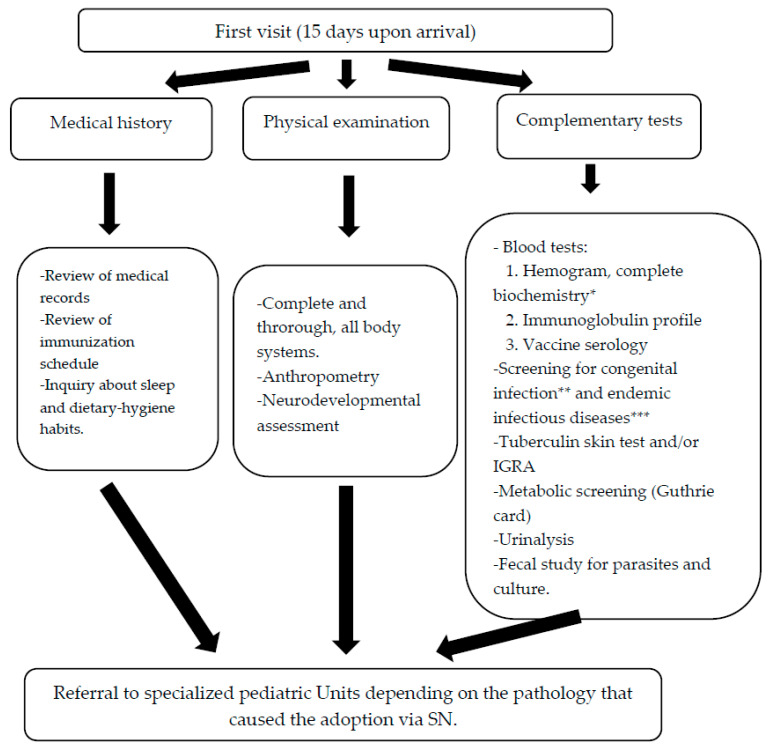
Management protocol for IA children with SN from the International Adoption Unit of the Pediatrics, Infectious and Tropical Diseases Service (Children’s Hospital La Paz—Carlos III). * Basic biochemistry, liver and kidney function. Consider ionogram, ferric profile, vitamin D, immunoglobulins G, A and M. Rickets screening: alkaline phosphatase, calcium, phosphates. ** CMV*, Toxoplasma*, HIV, hepatitis B, hepatitis C, syphilis. *** *Strongyloides, Schistosoma, Toxocara,* malaria.

The medical records were reviewed, and different variables were collected, starting at the pre-adoption visit and up to the sixth-month post-adoption visit. All children were managed according to a unified protocol, shown in Figure 1. Epidemiological variables such as age (in months), gender (male/female), country of origin (China, Vietnam, India, Ethiopia, Bulgaria), and situation before adoption (family versus institution) were collected through medical records and interviews with foster parents. All medical background was reviewed, including anthropometry (height, weight, and head circumference), actual and previous pathologies, immunization schedule in the country of origin, and laboratory test (hemoglobin in g/dl, serum iron in ng/mL, eosinophils in cells/µL), and/or serologies (human immunodeficiency virus [HIV], hepatitis B and C [HBV, HCV], syphilis and cytomegalovirus [CMV]) when performed. The pathology specified in the reports supplied by the country of origin was collected in the first consultation upon arrival in Spain. In addition, anthropometry and results of the complementary tests performed in our unit were analyzed, including serologies (HIV, HBV, HCV, syphilis, and CMV) and microbiological study for endemic parasites (*Schistosoma* spp., *Strongyloides stercoralis*, *Toxocara canis,* and/or malaria, according to local epidemiology). The results of the coproparasitological study, TB screening [tuberculin skin test (TST) and interferon-gamma release assay (IGRA)], and metabolic screening (using the Guthrie card) were included.

The hospital’s Ethics Committee approved the study (PI/4240) the 6 September 2018. No informed consent was required due to the retrospective design of the study. All data were collected on an anonymized data set. The data were used in compliance with the laws in Spain regarding data protection: Organic Law (LOPD) 3/2018 of 5 December, Protection of Personal Data.

### 2.2. Definitions

Low height or low weight for age and sex were considered when height or weight for age and sex were below 2 standard deviations (SD), according to WHO definitions [5]. Delay in pondero-statural growth (DPSG) was defined as a height and weight for age and sex below 2 SD [6]. Microcephaly was designated head circumference (HC) for age and sex below 2 SD [7].

Anemia was defined as a hemoglobin value < 10.5 g/decilitre (g/dL) in patients aged from 6 months to 2 years and <11.5 g/dL between 2 and 6 years of age [8].

Tuberculosis (TB) screening: tuberculin skin test was considered reactive above 10 millimeters of induration measured 72 h after inoculation. In those children under 3 years previously vaccinated with BCG [Bacillus Calmette-Guérin] and tuberculin skin test reactive, IGRA test was performed [9].

### 2.3. Statistical Analysis

We used descriptive statistics to summarize demographic and clinical characteristics. We reported qualitative data with absolute frequencies and percentages and quantitative data with medians with lower and upper quartiles (IQR). We compared the results of pre- and post-adoption reports using the chi-square or Fisher’s test for categorical variables and the Kruskal-Wallis test for continuous variables. The level of significance for all analyses was set at 0.05. Statistical analysis was performed with SPSS version 18.

## 3. Results

A total of 57 children (of whom 21 were female, 36.8%) were included, with a median age upon arrival in Spain of 27 months [IQR: 17–39]. Single-parent families corresponded to 48.2% of adopting families. Among the adoptees, Asian origin was predominant: 36 children (63.2%) from China, 18 (31.6%) from Vietnam, 1 (1.8%) from India, 1 (1.8%) from Ethiopia, and 1 (1.8%) from Bulgaria. In their country of origin, 73.7% lived in orphanages, and 5.3% with a foster family. One child (1.8%) had been hospitalized since birth. In 90% of the minors, the family history was unknown.

The pathologies reported in the pre- and post-adoption visits are compared in Table 1. Serologies reported in the pre- and post-adoption medical reports are compared in Table 2. The pathology that motivated the IA via SN was confirmed in 45 children (79%). Of the remaining 12, 8 were completely healthy children, and 4 had other minor pathologies upon arrival.

All patients with a previous diagnosis of congenital surgical malformation (23, 40.4%) as palatal malformation (12), congenital heart disease (6), or intervened digestive tract malformation (5 esophageal, ileal, or anal atresias) were confirmed (Table 1). Two patients reported a prior diagnosis of inguinal and umbilical hernias, neither observed in the post-adoption consultation.

Concerning patients with pre-adoption reported neurological pathologies, those were confirmed, and new diagnoses were added (two neurodevelopmental delays, one plagiocephaly), in addition to 10 cases of microcephaly not previously described.

According to the pre-adoptive report, out of six children with presumed congenital infection (three congenital syphilis, one hepatitis B, one hepatitis C, and one CMV infection), only one adequately treated congenital syphilis could be confirmed. All the parasitic infections were post-adoption diagnoses.

Regarding the dermatological pathology, two patients had in their reports benign vascular tumors that were the reason for adoption via SN (a dorsal hemangioma and a cervicobrachial hemangioma), both of which were confirmed on arrival. 

Four new cases of endocrinopathies were diagnosed during the medical assessment and follow-up (one case of hypothyroidism, one of precocious puberty, and two of methylcrotonylglycinuria). Additionally, another child presented on arrival with an alteration in the metabolism of acylcarnitines (an increase in the C3/C2 ratio in the metabolic screening) that was later improved, probably related to better nutrition after adoption.

Of the five patients with a previous diagnosis of respiratory disease (one bronchitis, two recurrent bronchospasms, one tracheitis, and one bronchopulmonary dysplasia), only one case of asthma was confirmed in the first post-adoption consultation.

Orthopedic pathology was reported in three children: two confirmed genu varum and a congenital dislocation of the hips that was not evident upon arrival. An undiagnosed genu varum was discovered in a patient with palatal malformation.

In the first post-adoption visit, 22.8% of the children were underweight, and 26.3% were low height, with 14% of adoptees meeting DPSG criteria compared to 1.8%, according to the pre-adoption reports.

The results of the most relevant complementary tests from the first visit are presented in Table 3 and Table 4, highlighting the infectious pathology in 17/57 (29.8%) children. Of 54 tuberculin skin tests performed, four (7.0%) were reactive. After completing the study with IGRA, tuberculosis infection (TBI) was ruled out in all of them.

A signed and stamped immunization schedule was provided by 91.2% of the children. However, only 7.7% of the patients in this document included all the vaccines recommended for age according to the immunization schedule of their country of origin.

## 4. Discussion

In this study in a referral unit in Spain, we confirmed that most of the children with special needs seen in our unit were of Asian origin (96.5%), as previously reported by other groups [4]. It was found that the pathologies that led to IA via SN were confirmed in most cases, especially when those were major surgical conditions (digestive tract, cardiac, or palatal malformations). Velopalatine malformations, such as cleft lip and palate, are one of the most prevalent conditions in our series, very frequently described in series of adopted children with SN [2]. Skin conditions are also frequently described in adoptees [10]. In our series, intestinal parasitosis and microcephaly were strongly underdiagnosed, according to pre-adoption reports. Other chronic conditions, such as four endocrinopathies, were also diagnosed upon arrival following the Spanish metabolic screening [11]. Thus, adoptive parents should know that the information provided by the institutions does not always offer complete accuracy, largely due to limited resources and healthcare unavailability in the country of origin [12].

There is a consensus that internationally adopted children should be evaluated by pediatricians with experience in the field. Most protocols agree on the importance of a thorough medical history upon arrival, an exhaustive physical examination, and complementary tests, followed by the completion of the immunization schedule [13,14]. In children with SN, the particular conditions of each individual case should be considered, and patients should be referred to specialists for proper multidisciplinary management [2,13].

Regarding oral health, children with SN show anxiety and uncooperative behavior during daily oral health or when oral treatments are needed. It has been proved that children with SN have worse oral health conditions than normal children [15]. Those with neurological or developmental disorders often have difficulty understanding the surrounding world and do not understand normal behaviors such as brushing their teeth [16]. Moreover, they require more assistance to maintain adequate oral health and have congenital developmental disorders that increase oral health inequality. They benefit from special dental clinics, where health providers have appropriate skills to evaluate and treat them [17].

In terms of anthropometric measurements, according to the pre-adoption reports, only one patient in our series had DPSG; however, after evaluation in our unit, 14% were diagnosed with DPSG. The etiology of growth delay is multifactorial, and it might be related to insufficient nutritional intake and emotional disorders, both common factors among adopted children. Especially worrisome is the high rate of underdiagnosed microcephaly (17.5%) in the post-adoption consultation. Microcephaly might also be related to emotional and attachment disorders, but differential diagnosis is wide, and some underlying conditions might imply neurocognitive impairment. Long-term follow-up will be needed in order to achieve a final diagnosis in most of the children included in this study. The high prevalence of microcephaly and underweight in children from IA has already been described in previous series and interpreted as a sign probably related to direct consequences of the medical conditions and the emotional depletion suffered [14].

Psychological support is essential to improve language, communication, and social skills and to prevent school failure as they are known to be at risk compared to non-adopted children of the same socioeconomic profile [18,19,20]. Many of these children develop learning difficulties in the future. Having a diagnosis is crucial for them, as it improves their psychological and scholastic well-being [21]. Rehabilitation therapies are also important for children with special learning disorders or cerebral palsy [22]. Music can also improve language skills and be an educational tool for children with SN with promising results [23]. A Taiwanese study enrolled 81 children with SN aged 24–60 months in order to evaluate the impact of a holistic music educational approach [24]. After 16 weeks, children showed a significant improvement in the learning assessment indicators such as language comprehension, language expressiveness, self-control, interpersonal relationships, social skills, and physical movement. In our unit, psychological support is offered to all children and their families, focusing overall on those with neurological disorders or whose mothers had an alcohol/drugs consumption history.

As described in other series [25,26,27,28], the most prevalent infections in our patients were intestinal parasitosis (8/57), followed by a positive serology result for schistosomiasis (7/40), strongyloidiasis (3/49), or toxocariasis (3/49). Upon arrival in the new home country, the screening of infectious diseases must be individualized and tailored to the origin and previous living conditions. Most parasitoses are linked to poor hygienic-sanitary conditions and can be treated [2,29]; however, intestinal parasitosis in early childhood has been associated with long-term nutritional problems and neurocognitive development [30]. Congenital infections were uncommon: out of the six children previously diagnosed with congenital infection, only one case of correctly treated congenital syphilis was confirmed. These findings highlight the importance of performing complete infectious diseases screening to confirm presumptive diagnoses and rule out previously non-diagnosed pathologies [27]. Among other infectious diseases, screening for tuberculosis in adoptees is essential since they come primarily from endemic areas [31]. There is controversy about the convenience of the different diagnostic tests [32,33]. In our study, the absence of TBI is striking, different from what is described in other series [34]. However, it may be explained by the younger age of our patients and/or by the isolation of the minors with SN, possibly with little contact with other institutionalized children.

Many authors have reported how children with chronic conditions are often not adequately vaccinated in different contexts. In our series, only 7.7% had a complete immunization schedule for their age and country of origin. Additionally, the immunization record is not always accurate, and the vaccines received in their country of origin are minimal [35]. It is always advisable to adapt the immunization of these children with the necessary doses depending on their age [36,37].

Our study has several limitations, such as the retrospective design, with the constraints that this entails. Moreover, most reports are concise or just summaries of the previous diagnosis arranged by non-medical staff. Data regarding personal and family history are usually absent. In other cases, reports can be challenging to interpret due to poor translation [3]. However, we present one of the most extensive series of internationally adopted children with special needs, describing their clinical and epidemiological characteristics.

## 5. Conclusions

In our series, the pathologies stated in the pre-adoption reports of children with special needs were confirmed in 80% of cases. The most frequently reported diseases were congenital surgical malformations and mild neurological and hematological conditions. However, upon arrival, height and growth delays, microcephaly, and infectious diseases were commonly underdiagnosed.

Internationally adopted children with special needs constitute a challenge for both healthcare professionals and adoptive parents, who must be aware of the difficulties that may arise. Clinical and psychological support should be provided throughout the whole adoption process, ideally in specialized units with experienced multidisciplinary teams able to provide comprehensive care to these minors.

## Figures and Tables

**Table 1:** Pre- and post-adoption results regarding pathologies reported in the respective medical reports.

	Pre-Adoption Results, N (%)	Post-Adoption Results, N (%)	
General Pathology	57/57 (100)	57/57 (100)	*p*
**Palatal malformation**	12 (21.0)	12 (21.0)	1
**Cardiopathy**	9 (15.8)	12 (21.0)	0.468
Surgically corrected congenital cardiopathies	6 (10.5)	6 (10.5)	1
Interatrial communication	1 (1.8)	0	-
Patent foramen ovale	2 (3.5)	3 (4.8)	
Other	0	4 (7.0)	-
**Digestive pathology**	9 (15.8)	6 (10.5)	0.405
Surgically corrected digestive tract malformations	5 (8.8)	5 (8.8)	1
Abdominal wall malformations	2 (3.5)	0	
Digestive problems	3 (5.3)	1 (1.8)	-
**Neurological pathology**	14 (24.6)	22 (38.6)	0.106
Neurodevelopmental delay	4 (7.0)	6 (10.5)	
Sensorineural deafness	2 (3.5)	2 (3.5)	1
Intracranial hypertension	1 (1.8)	1 (1.8)	1
Alterations in craniofacial morphology			
-Plagiocephaly	7 (12.3)	8 (14.0)	
-Macrocephaly	1 (1.8)	1 (1.8)	1
-Microcephaly	0	10 (17.5)	
**Infectious pathology**	7 (12.3)	17 (29.8)	**0.021**
Intestinal parasitosis	0	8 (14.0)	-
Systemic parasitosis *	0	11 (19.3)	-
Congenital infections	6 (10.5)	1 (1.8)	
Other	1 (1.8)	1 (1.8)	
**Dermatological pathology**	6 (10.5)	8 (14.0)	0.568
Hemangioma	2 (3.5)	2 (3.5)	1
Atopic dermatitis	3 (5.3)	3 (5.3)	1
Scabies	1 (1.8)	1 (1.8)	1
Vitiligo	0	1 (1.8)	-
Allopecic plaque in scalp	0	1 (1.8)	-
**Suspected endocrinopathy**	-	5 (8.9)	-
**Respiratory pathology**	5 (8.8)	1 (1.8)	0.093
**Orthopedic pathology**	3 (5.3)	3 (5.3)	1
**Visual anomalies**	4 (7.0)	8 (14.0)	0.222
Visual acuity alteration	3 (5.3)	7 (12.3)	
Retinopathy of prematurity	1 (1.8)	1 (1.8)	1
**Hematological pathology**	14 (22.6)	4 (7.0)	**0.012**
Anemia not otherwise specified	9 (15.8)	2 (3.5)	
Thalassemia	4 (7.0)	2 (3.5)	
Jaundice	1 (1.8)	0	-
**Delay in pondero-statural growth**	1 (1.8)	8 (14.0)	**<0.05**

* Strongyloidiasis, schistosomiasis, toxocariasis.

**Table 2:** Pre- and post-adoption results regarding serologies reported in the respective medical reports.

	Pre-Adoption Results, N (%)	Post-Adoption Results, N (%)
Serologies (congenital infections)	56 (98.3)	57 (100)
Anti-HIV antibodies (tested)	53 (93.0)	57 (100)
Positive results	0	0
Syphilis (RPR and/or VDRL tested)	51 (91.1)	57 (100)
Positive results	2 (3.9)	1 (1.8)
HBs antigen (tested)	55 (96.5)	57 (100)
Positive results	1 (1.8)	0
Anti-VHC antibodies (tested)	3 (5.3)	57 (100)
Positive results	1 (33.3)	0

**Table 3:** Complementary blood tests performed after first visit upon arrival.

Laboratory tests	
Hemoglobin	Median 13 mg/dL, IQR (12-14)
Anemia	4/56: 7.1%
Iron	Median 29 ug/dL, IQR (20-40)
Iron deficiency (≤ 15 ng/mL)	9/53 (17%)
Eosinophils	Median 195 cél/µL, IQR (147-330)
Eosinophilia (> 500/mL)	7/56 (12.5%)
Metabolic screening	
Normal	41/44 (93.2%)
Non conclusive	2/44 (4.5%)
Increase in C3/C4 ratio	1/44 (2.3%)

**Table 4:** Microbiologic tests performed after first visit upon arrival.

Microbiology	
Serologies	
*Schistosoma* spp.	7/40 (17.5%)
*Strongyloides stercoralis*	3/49 (6.1%)
*Toxocara canis*	3/49 (6.1%)
Positive *Plasmodium* antigen CMV IgG	0/10 24/28 (85.7%)
Stool studies	
Parasites detected *	7/56 (12.5%)
Ag *Cryptosporidium* o *Giardia lamblia*	1/37 (2.7%)
Tuberculosis screening	
Positive tuberculin skin test	4/54 (7.4%)
IGRA	0/5

* Strongyloidiasis, schistosomiasis, toxocariasis.

## Data Availability

The data presented in this study are available on request from the corresponding author.
